# Clinical image—postpartum clitoral epidermoid cyst

**DOI:** 10.1007/s00192-022-05177-7

**Published:** 2022-04-05

**Authors:** Ebunoluwa Oluwaferanmi, Swati Jha

**Affiliations:** Department of Urogynaecology, Sheffield Teaching Hospitals, Level 4 Jessop Wing, Tree Root Walk, Sheffield, S10 2SF UK

**Keywords:** Clitoral cyst, Epidermoid cyst, Postpartum, Excisional biopsy

## Introduction

Clitoral epidermal cysts are benign lesions that can be excised surgically and carry good prognosis after treatment. They are usually slowly growing tumors that arise due to invagination of epidermis into dermis either spontaneously or following trauma. The differential diagnosis of clitoral cysts depends on the age of presentation. When these cysts present in children, they mimic clitoromegaly and can be confused with virilization due to hyperandrogenic states. Non-hormonal differential diagnosis includes neurofibromatosis (NF) and nevus lipomatous cutaneous superficialis.

We present images of a female who developed this cyst post-partum.

## Case report

A 31-year-old woman presented to our team 18 months post-cesarean section after her first child, with a slowly but progressively enlarging clitoral lump first noticed about a month postpartum. The cesarean was uneventful and not associated with any trauma. She had not undergone any form of trauma to the clitoral area such as female genital mutilation (FGM) previously.

**Fig. 1:**
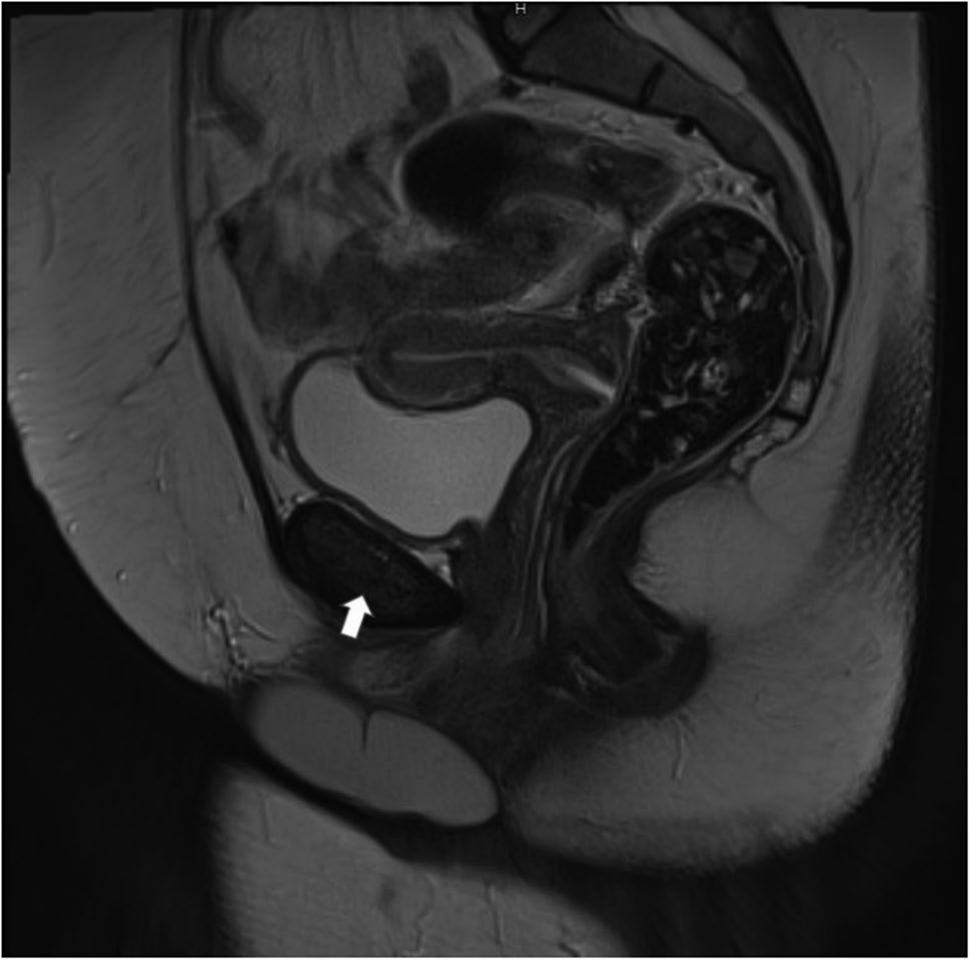
MRI sagittal view of clitoral cyst

**Fig. 2:**
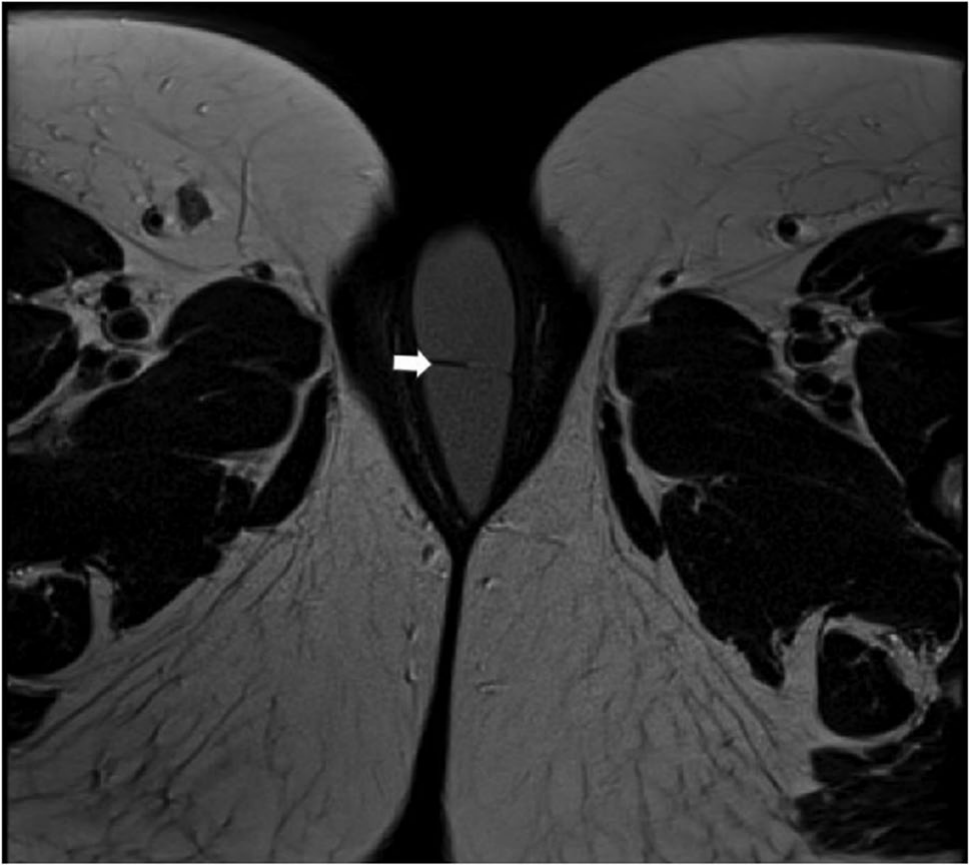
MRI transverse view of clitoral cyst

Perineal examination was normal except for an 8 cm, soft and fluctuant, mildly tender clitoral swelling with no evidence of involvement or obstruction to the urethral and vaginal openings. Magnetic resonance imaging (MRI) pelvis (Figs. 1 and 2) confirmed an isolated clitoral cyst with no connection to the pelvis or surroundings organs. The cyst was excised under general anesthesia while avoiding the areas of innervation of the clitoris and surgical reconstruction done for cosmesis. The patient developed a secondary infection on the 2nd postoperative day, which was treated with antibiotics.

**Fig. 3:**
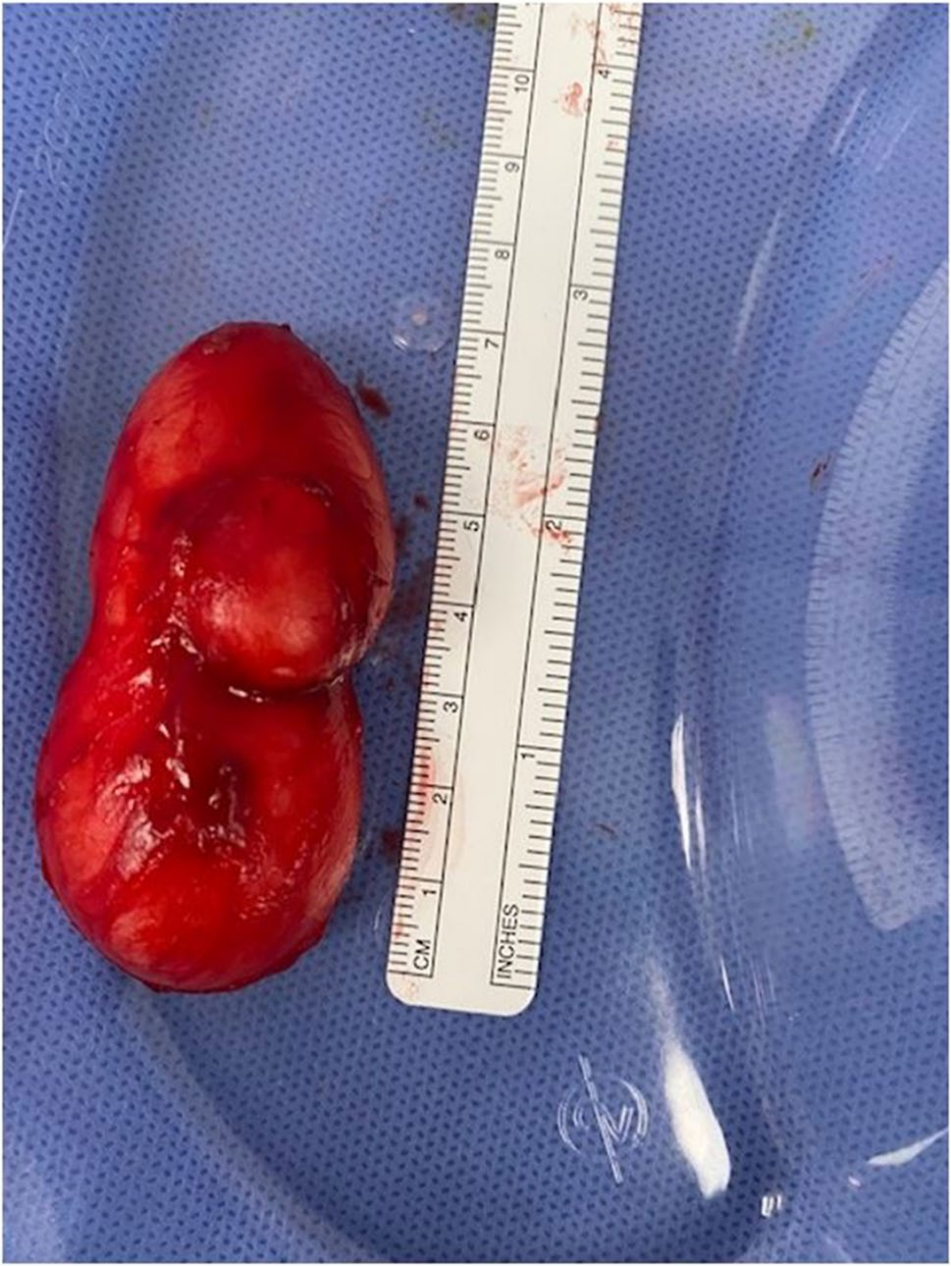
Excised clitoral cyst sent for histology

Histology showed a thin-walled, lobulated tense cyst containing beige creamy material, measuring 65 × 28 × 32 mm, confirmed to be epidermal cyst (Fig. 3). Postoperative outcome demonstrated a normal appearance of the clitoris.

Clitoral epidermal cysts are very rare with only a few cases reported in literature [1, 2] including one report described in a pregnant female [3]. Concerns with removal include the impact on sexual function and cosmesis, and patients should be forewarned of these risks.
